# Selective serotonin reuptake inhibitor, fluoxetine, impairs E-cadherin-mediated cell adhesion and alters calcium homeostasis in pancreatic beta cells

**DOI:** 10.1038/s41598-017-03747-0

**Published:** 2017-06-14

**Authors:** Huang-Yu Chang, Shu-Ling Chen, Meng-Ru Shen, Mei-Lang Kung, Lee-Ming Chuang, Yun-Wen Chen

**Affiliations:** 1 Department of Pharmacology, College of Medicine, National Cheng Kung University, Tainan, Taiwan; 2 Department of Obstetrics and Gynecology, College of Medicine, National Cheng Kung University, Tainan, Taiwan; 3 Advanced Optoelectronic Technology Center, College of Engineering, National Cheng Kung University, Tainan, Taiwan; 4 Department of Chemistry, National Sun Yat-Sen University, Kaohsiung, Taiwan; 5 Department of Internal Medicine, National Taiwan University Hospital, Taipei, Taiwan; 6 Department of Medicine, National Taiwan University Medical College, Taipei, Taiwan

## Abstract

Selective serotonin reuptake inhibitors (SSRIs) are the most commonly prescribed drugs for mood disorders. Long term use of SSRIs is associated with an increased risk of diabetes, but the underlying mechanism(s) remains elusive. E-cadherin-mediated cell-cell adhesion and elevated [Ca^2+^]_i_ are important for insulin release and pancreatic β cell functions. This study aims to investigate whether a SSRI, fluoxetine (Prozac), induces pancreatic β cell dysfunction through affecting E-cadherin and/or [Ca^2+^]i. Here we show that fluoxetine significantly reduces glucose stimulated insulin secretion (GSIS). MIN6 cells, an established murine immortalized β cell line, form smaller colonies of loosely packed cells with reduced cell-cell contact after fluoxetine treatment. Immunofluorescence staining reveals that fluoxetine increases cytoplasmic accumulation of E-cadherin and reduces the membrane-localized E-cadherin probably due to increase of its endocytosis. Fluoxetine inhibits spreading of β cells on E-cad/Fc coated slides and also disrupts E-cadherin-mediated actin filaments. Additionally, fluoxetine significantly suppresses endoplasmic reticulum (ER) calcium release and store-operated calcium entry (SOCE) activation, probably through reduction of ER calcium storage and inhibition of stromal interaction molecule 1 (STIM1) trafficking. These data suggest that exposure to fluoxetine results in impaired β cell functions, occurring in concert with reduction of E-cadherin-dependent cell adhesion and alterations of calcium homeostasis.

## Introduction

Patients with major depressive disorder (MDD) have a higher incidence of type 2 diabetes mellitus (T2DM) when compared to the general population^1, 2^. Although the underlying mechanism(s) involved in the relationship between T2DM and MDD is not fully understood, recently a growing number of studies indicate that long-term use of SSRIs constitutes to a major risk factor for impaired glucose homeostasis and development of T2D^3–5^. Similarly, a recently population-based, nested case-control study in Taiwan showed a 20% increased risk of diabetes for patients with long-term antidepressant treatment for two or more years^6^. Despite these findings, little is known about the direct pathophysiology of SSRIs on pancreatic β cell functions. Early studies demonstrated that administration of fluoxetine and fluvoxamine induced hyperglycemia in rodents^7, 8^. Isaac *et al*. showed that acute treatment of MIN6 β cells with sertraline (30 μM) inhibited glucose-stimulated insulin secretion (GSIS) and resulted in the inhibition of insulin action^9^. Recent work by De Long *et al*. reported that chronic treatment of INS-1E β cells with fluoxetine (1 μM) impaired GSIS and increased the production of reactive oxygen species (ROS) resulting in damage to mitochondrial function^10^. Moreover, fluoxetine-induced impairment of GSIS observed in β cell lines may be independent of accumulation of extracellular 5-HT level^11^.

Cell communication and synchronization are necessary for fully functional pancreatic islets due to composed of diverse cells, such as glucagon-producing alpha cells and insulin-producing β cells^12, 13^. Adherens junction mediated by E-cadherin and N-cadherin supports cell adhesion and stabilizes the structure of the pancreas^12^; this E-cadherin-mediated interaction is required for proper pancreatic nutrient-sensing response^14, 15^. Consistently, insulin secretion from human β cells is highly dependent on cell-cell contact^16^. An elegant study using chimeric proteins made of functional cadherin ectodomains fused to the Fc fragment of immunoglobulin showed the E-cadherin-mediated cell adhesion is essential for β cells viability and GSIS^17, 18^. RNAi mediated silencing of E-cadherin in MIN6 cells and isolated islets decreases GSIS^19^. All the consequences indicate that E-cadherin is necessary for the functions of β cells, especially insulin secretion in response to glucose stimulation.

Modulation of cytosolic free calcium concentration ([Ca^2+^]_i_) is an important for signal transduction involved in fundamental cellular functions, such as proliferation, migration, gene regulation, and apoptosis in various cell types^20, 21^. These changes either reflect alterations in calcium (Ca^2+^) fluxes or result from mobilization of intracellular Ca^2+^ stores^20, 22^. Elevation of [Ca^2+^]_i_ is critical for secretagogue-induced insulin secretion in pancreatic islet cells^23^. The depletion of the ER Ca^2+^ stores in non-excitable cells is coupled to the activation of Ca^2+^ influx, a process referred to as store-operated Ca^2+^ entry (SOCE or capacitative Ca^2+^entry, CCE)^24^; SOCE also presents in excitable cells, such as the insulin-secreting β cells^25^. Two proteins, STIM (stromal-interaction molecule) and Orai, are the molecular identities responsible for SOCE activation. STIM1 protein functions as an ER Ca^2+^ sensor, which clusters proximally to plasma membranes to activate Orai1 during ER Ca^2+^ depletion^26, 27^. Accumulating evidence indicates that altered SOCE is associated with diabetes complication^28, 29^. Studies show that depletion of intracellular Ca^2+^ stores in β cells activate SOCE^25, 30^. Recently, Sabourin *et al*. demonstrated that Orai1 and TRPC1, which form the SOCs regulated by STIM1, are implicated in insulin secretion in β cells^31^.

In the present study, we sought to examine whether fluoxetine impairs insulin secretion from pancreatic β cells through affecting E-cadherin-mediated cell-cell adhesion and/or [Ca^2+^]i. Here, we demonstrate that fluoxetine treatment of pancreatic β cells results in both reduction of cell-cell adhesion and alteration of calcium homeostasis, leading to the decrease of GSIS and impaired pancreatic β-cell functions.

## Results

### Fluoxetine treatment alters cell morphology, and reduces cell-cell adhesion

**Figure 1:**
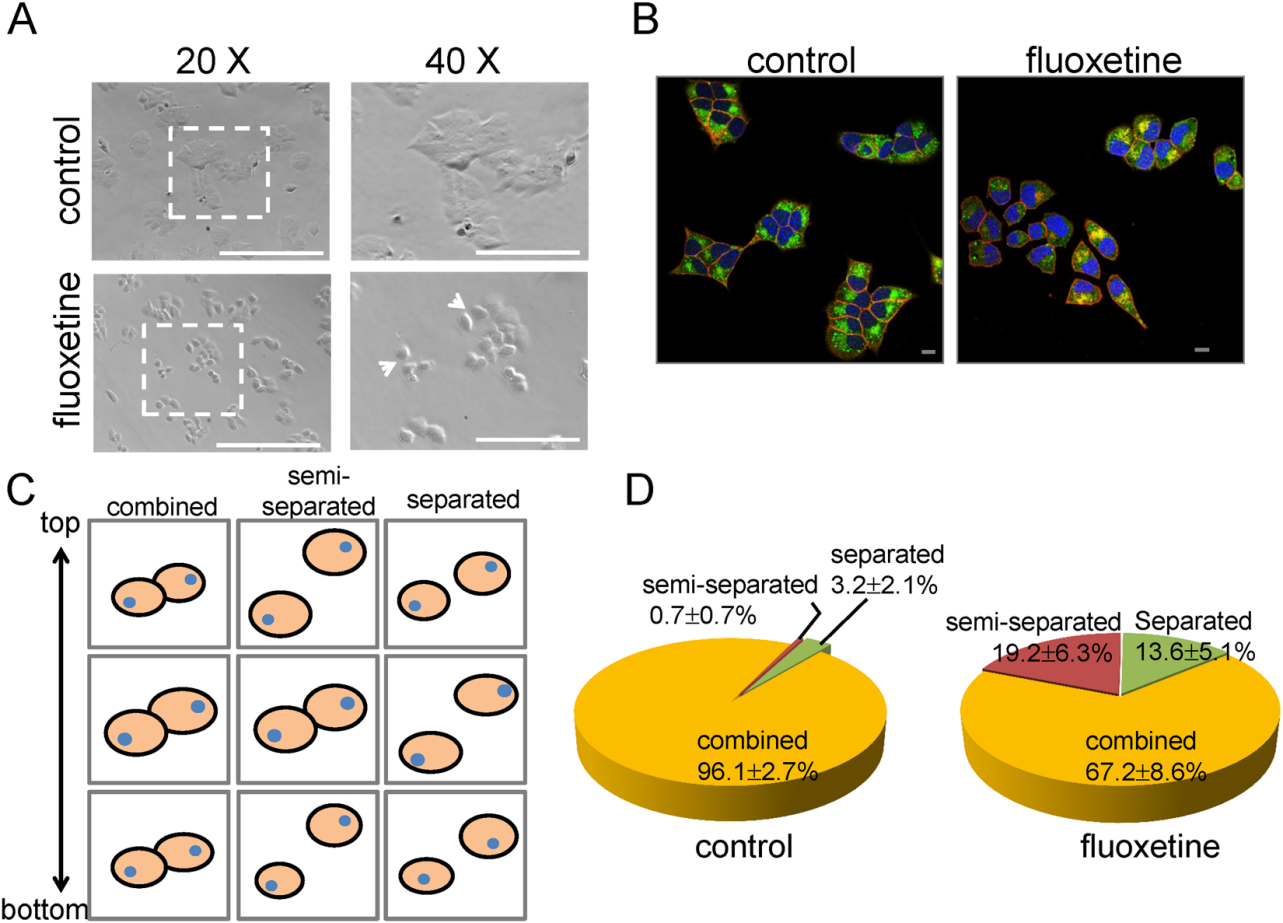
Fluoxetine alters cell morphology, and reduces cell-cell adhesion. (**A**) After 3-hour fluoxetine (30 μM) treatment, MIN6 cells were observed under an inverted fluorescence microscope (Evos). The white arrows indicate reduction of cell-cell adhesion. Scale bar, 100 μm. The representative images were from at least three independent experiments. (**B**) After 3-hour incubation with or without fluoxetine (30 μM), MIN6 cells were fixed and then immuno-stained with Alexa 488 (green) for E-cadherin, Alexa 594 (red) for β-catenin and Hoechst 33258 (blue) for nucleus. The images were captured by using confocal microscope (Olympus, MPE). Scale bar, 10 μm. The representative images were from at least three independent experiments. (**C**) Schematic diagram defines three characteristics of cell contact. Cells were categorized by how close they contact to each other at different z-sections. Cell junction was fully continuous from top to bottom (“combined” cells), partially lost at the top and bottom (“semi-separated” cells) or totally lost from top to bottom (“separated” cells). (**D**) Quantitative analysis for the percentage of each contact type of MIN6 cells treated with or without fluoxetine. Each value represents mean ± SEM of at least 600 individual cells.

To investigate the effects of fluoxetine on β cell function, MIN6, an established murine immortalized β cell line was employed as an *in vitro* model^32^. Cells were incubated with fluoxetine, a widely used SSRIs^33^, for 3 h. Our results showed that fluoxetine (30 μM) had no effect on cell proliferation and cell viability (Fig. S1A,B); however, it significantly inhibited GSIS (Fig. S1C). Next, we sought to understand the cellular and molecular events underlying this deleterious effect of fluoxetine on insulin secretion. Cell-cell adhesion plays an important role in regulating GSIS from pancreatic β cells^16, 18^, so next we examined whether fluoxetine can affect cell morphology, and cell-cell adhesion. Our results showed that MIN6 cells grew in tightly packed colonies with close cell-cell contact in the control group, while cells formed smaller colonies of loosely packed cells with reduced cell-cell contact in the fluoxetine-treated group (Fig. 1A). To assess the role of adhesion molecules in mediating the alteration in cell morphology, MIN6 cells were immuno-stained with Alexa 488 (green) for E-cadherin and Alexa 594 (red) for β-catenin (Fig. 1B). We found control group with adjacent cells within each colony shared common boundaries demarcated by E-cadherin, but E-cadherin was reduced at area of cell contact and cell dispersed after fluoxetine treatment (Fig. 1B). Here we defined three characteristics of cell populations from our confocal images by performed z-section from top to bottom of cells (Fig. 1C). Combined cells stood for cells stick together at each stage, while separated cells represented that cells were totally disconnected from the top to bottom. Interestingly, there were some cells being associated to each other at the middle stage, but separated at the top and bottom stage. We defined this population as semi-separated cells. Quantification of these three characteristics of cell populations from confocal images stage-by-stage, as shown in Fig. 1D, 96.1 ± 2.7% of control cells combined to other cells, but only 67.2 ± 8.6% of fluoxetine-treated cells remained combined. The results indicated that fluoxetine altered cell morphology correlated with a loss of cell-cell adhesion.

### Fluoxetine alters the structure of adherens junction and the distribution of E-cadherin

**Figure 2:**
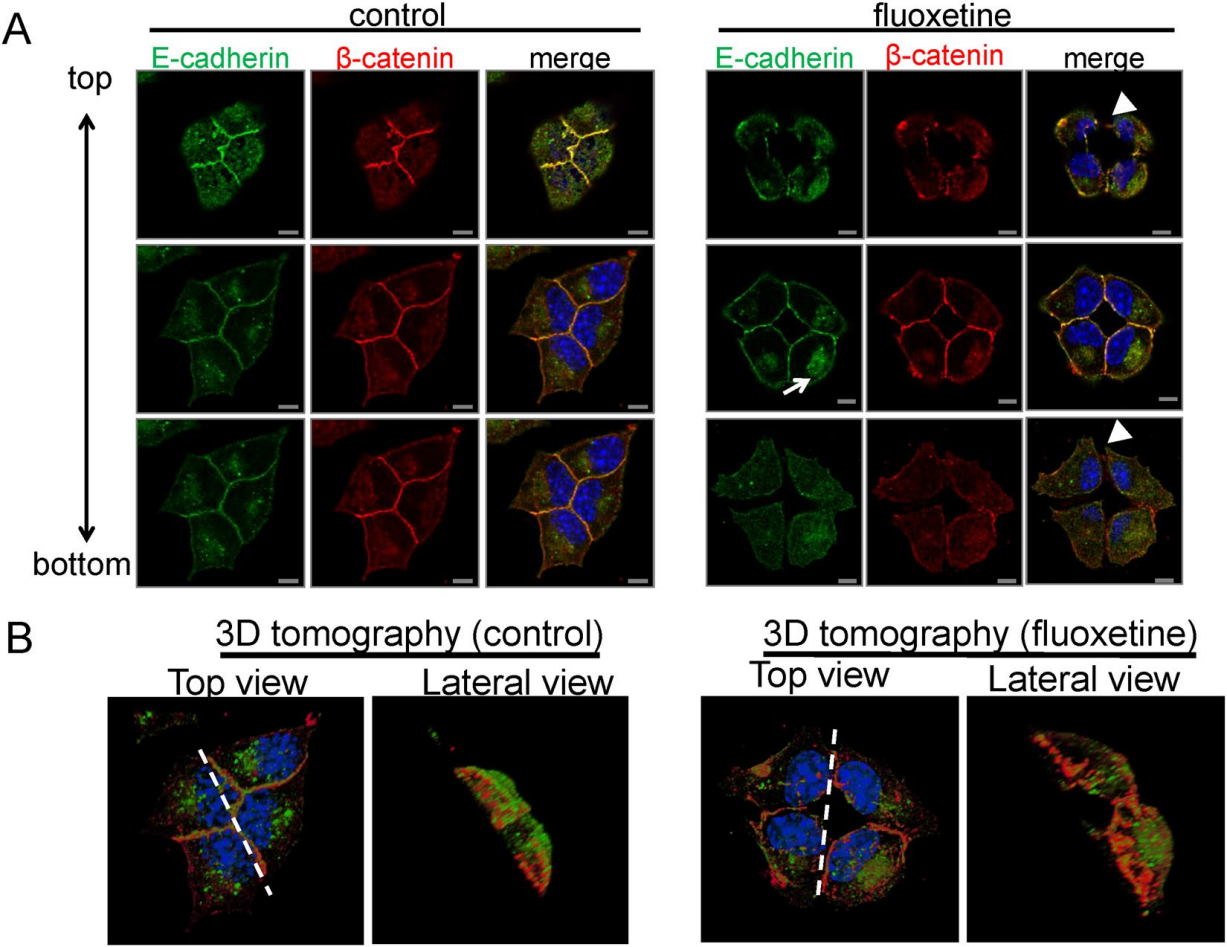
Fluoxetine alters the structure of adherence junction and the subcellular distribution of E-cadherin. After 3-hour treatment with or without fluoxetine (30 μM), MIN6 cells were fixed and then immune-stained with Alexa 488 (green) for E-cadherin, Alexa 594 (red) for β-catenin and Hoechst 33258 (blue) for nucleus. The images were captured by confocal microscope (Olympus, MPE). Scale bar, 10 μm. (**A**) Representative confocal images showing the distribution of E-cadherin and β-catenin at different z-sections. White arrowheads indicate separated cells. Arrows indicates the aggregation of E-cadherin in cytoplasm. The representative images were from at least three independent experiments. Scale bar, 5 μm. (**B**) The 3D images of E-cadherin and β-catenin distribution were visualized by the Avizo software from at least 30 slices of confocal images.

Adherens junction is the most important structure to maintain cell-cell adhesion. E-cadherin connects neighboring cells at outer membrane^34^, and is regulated by cytosolic protein β-catenin^35, 36^. To investigate whether fluoxetine affects the adherens junction, E-cadherin and β-catenin were immuno-stained and visualized by confocal microscope. MIN6 cells treated with fluoxetine did not share a common boundary with their neighbors and were physically separated by distinctly visible gaps between E-cadherin demarcated membranes, indicating reduced cell-cell adhesion (Fig. 2A). The 3D image constructed from confocal images clearly shows the location of E-cadherin expression at cell junction was altered by fluoxetine treatment (Fig. 2B). To our surprise, immunofluorescence staining showed that E-cadherin appeared to accumulate in cytosol after fluoxetine treatment (Fig. 2A). Furthermore, our *in vivo* data revealed that the pancreatic islets from mice treated with fluoxetine showed a decrease in junctional E-cadherin (Fig. S2)

### Fluoxetine induces E-cadherin accumulation in Golgi apparatus, but not ER

**Figure 3:**
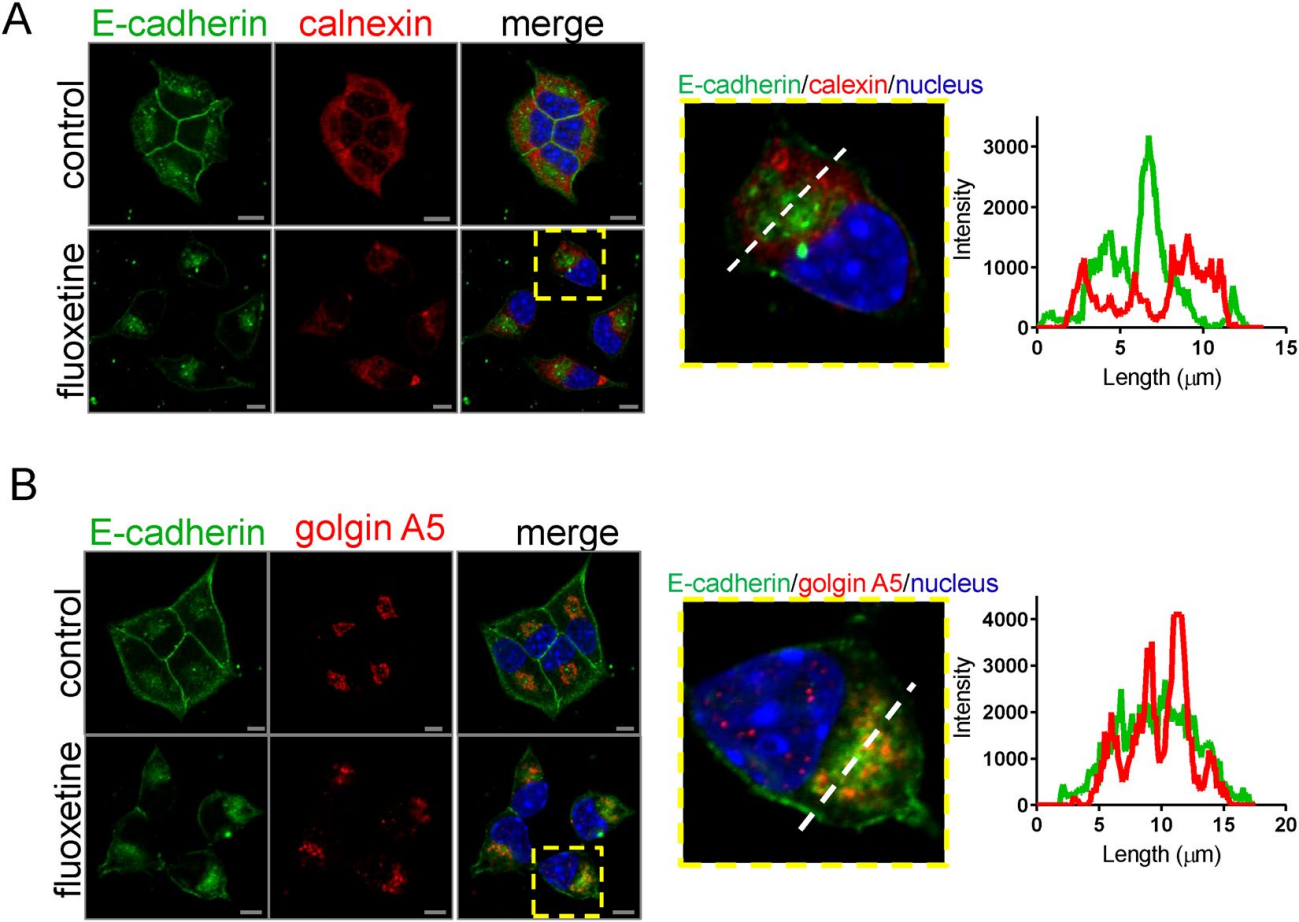
Fluoxetine induces E-cadherin accumulation in Golgi apparatus, but not ER. After 3-hour incubation with or without fluoxetine (30 μM), MIN6 cells were fixed and then immunostained. (**A**) (left) Representative confocal images show the subcellular localization of E-cadherin (green) and an ER marker calnexin (red). Nuclei were stained with Hoechst 33258 (blue). Scale bar, 5 μm. (middle) Image represents the enlargement of the areas indicated by rectangles in the whole-cell images in panel A. (right) Graph shows quantitative analysis of fluorescence intensity (F.I.) along the dashed line in panel A by FV 1000 software. (**B**) (left) Representative confocal images represent the subcellular localization of E-cadherin (green) and a Golgi marker Golgin A5 (red). Nuclei were stained with Hoechst 33258 (blue). Scale bar, 5 μm. (middle) Image represents the enlargement of the areas indicated by rectangles in whole-cell images in panel B. (right) Graph shows quantitative analysis of fluorescence intensity (F.I.) along the dashed line in panel B by FV 1000 software.

Translated from ER, pro-E-cadherin is transported to the Golgi apparatus for protein trimming/maturation and then the mature E-cadherin is delivered to the plasma membrane^37^. Since fluoxetine induced accumulation of cytosolic E-cadherin in treated MIN6 cells (Fig. 2A), we next sought to identify the cellular compartment where E-cadherin accumulates in the cytosol under fluoxetine treatment. Our immunostaining data showed that accumulated cytosolic E-cadherin did not co-localize with calnexin, an ER marker (Fig. 3A), suggesting that cytosolic E-cadherin did not accumulate in ER. This is confirmed by the fluorescence intensity profile, showing that the peak of E-cadherin expression did not overlap with that of calnexin (Fig. 3A). Golgin subfamily A member 5 (Golgin A5), which is responsible for vesicle tethering and docking at Golgi apparatus, serves as a marker of the Golgi apparatus. Immunofluorescence staining demonstrated that localization of Golgin A5 (red) was consistent with E-cadherin staining (green) under fluoxetine treatment, suggesting that cytosolic E-cadherin accumulated in the Golgi (Fig. 3B). Additionally, fluorescence intensity profile showed that the peak of Golgin A5 was correlated with that of E-cadherin (Fig. 3B). Thus, our data indicated that the fluoxetine induced the accumulation of E-cadherin in the Golgi apparatus, but not ER.

### Fluoxetine reduces the membrane-localized E-cadherin by increasing E-cadherin endocytosis

**Figure 4:**
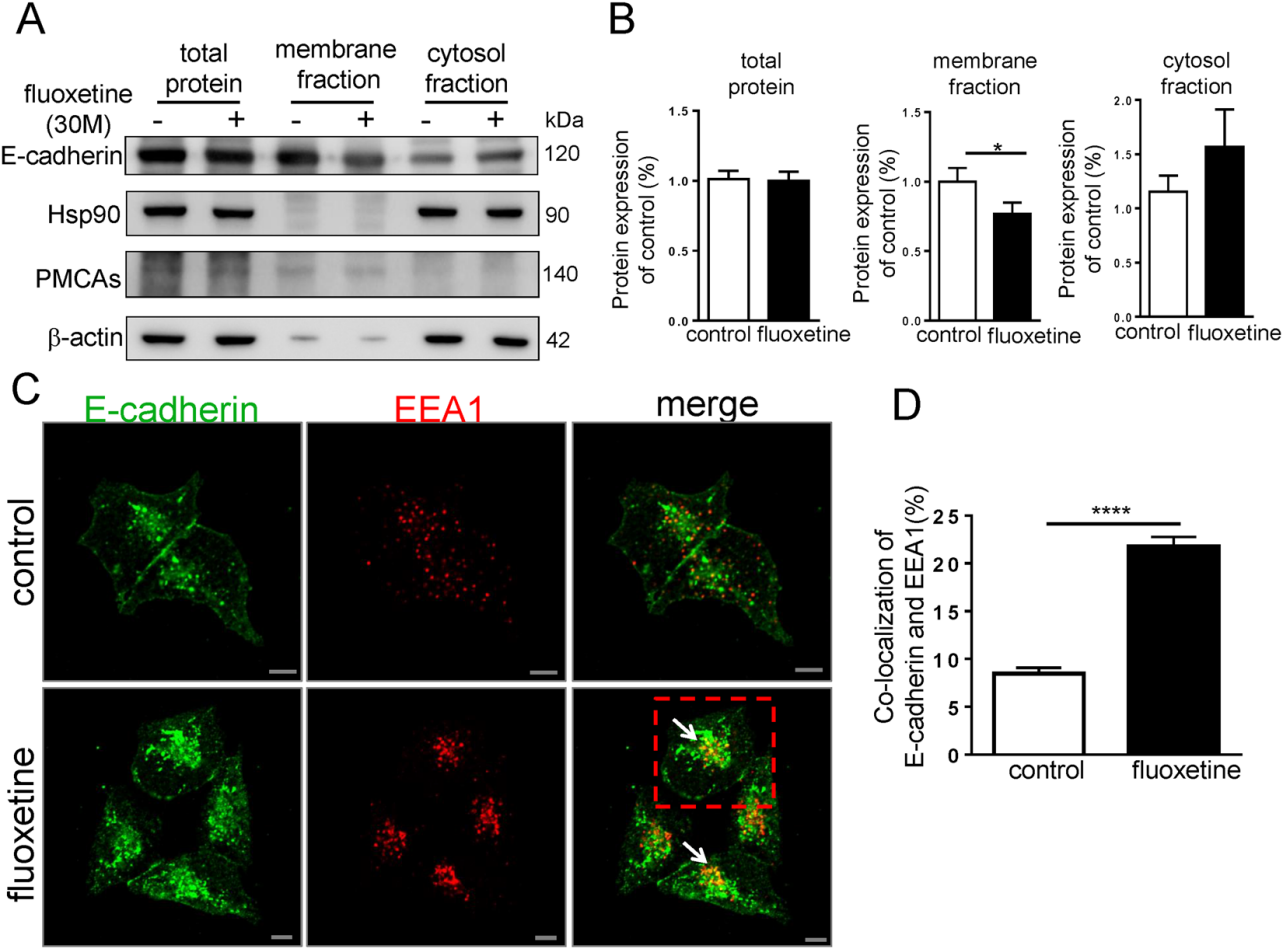
Fluoxetine reduces the membrane-localized E-cadherin and increases E-cadherin endocytosis. (**A**) After 3-hour incubation with or without fluoxetine (30 μM), cellular proteins were harvested by using a biotin-surface protein isolation assay, and then analyzed by western blotting. E-cadherin in total lysates, membrane fractions and cytosol fraction are shown. β-actin was used as a loading control for total protein. Hsp90 (heat shock protein 90) was used as a loading control for cytosol fraction. PMCA (calcium pump pan PMCA ATPase) was used as a loading control for membrane fraction. The representative data was from at least 3 independent experiments. Cropped blots have been presented. Full length blots are presented in Supplementary Fig. S6. (**B**) Quantitative analysis of total E-cadherin, membrane E-cadherin and cytosolic E-cadherin expression. Values (means ± SEM) are from three independent experiments. *P < 0.05 (**C**) After 3-hour fluoxetine (30 μM) treatment, MIN6 cells were fixed and then stained. The representative confocal images show the localization of E-cadherin (green) and EEA1 (red) in MIN6 cells. Nuclei were stained with Hoechst 33258 (blue). White arrows indicate the co-localization of E-cadherin and EEA1. Scale bar, 5 μm. The representative images were from three independent experiments. (**D**) Co-localization ratio between E-cadherin and EEA1 with pixel-by-pixel analyses. Each value represents mean ± SEM from at least 30 different cells from three independent experiments. ***P < 0.001.

Mature E-cadherin mediates adhesion function at the cell junction and its membrane localization is essential for adhesion activity^38, 39^. To examine whether fluoxetine has an impact on cell surface expression of E-cadherin, we performed a cell surface protein isolation assay. Our data showed fluoxetine-treated cells displayed decrease E-cadherin expression at the cell surface when compared to control cells (Fig. 4A and B). Of note, the total amount of E-cadherin remained unchanged after fluoxetine treatment (Fig. 4A and B). The quantitative data showed a reduction of 33% in membrane-localized E-cadherin after fluoxetine treatment (Fig. 4B). Adherens junction is a highly dynamic structure^40^. The rate of endocytosis and exocytosis of E-cadherin regulates the activity of cell-cell adhesion^41^. We further asked whether the intracellular accumulation of E-cadherin in fluoxetine-treated cells was caused by an increase of its endocytosis. An early endocytosis marker, early endosome antigen1 (EEA1) was employed to test the endocytosis of E-cadherin. Fluoxetine increased co-localization of cytosolic E-cadherin and EEA1, which was not observed in control condition (Fig. 4C). The quantitative data of the co-localization of EEA1 and E-cadherin suggested that fluoxetine significantly increased the endocytosis of E-cadherin (Fig. 4D).

### Fluoxetine inhibits the spreading of β cells attached to E-cad/Fc

**Figure 5:**
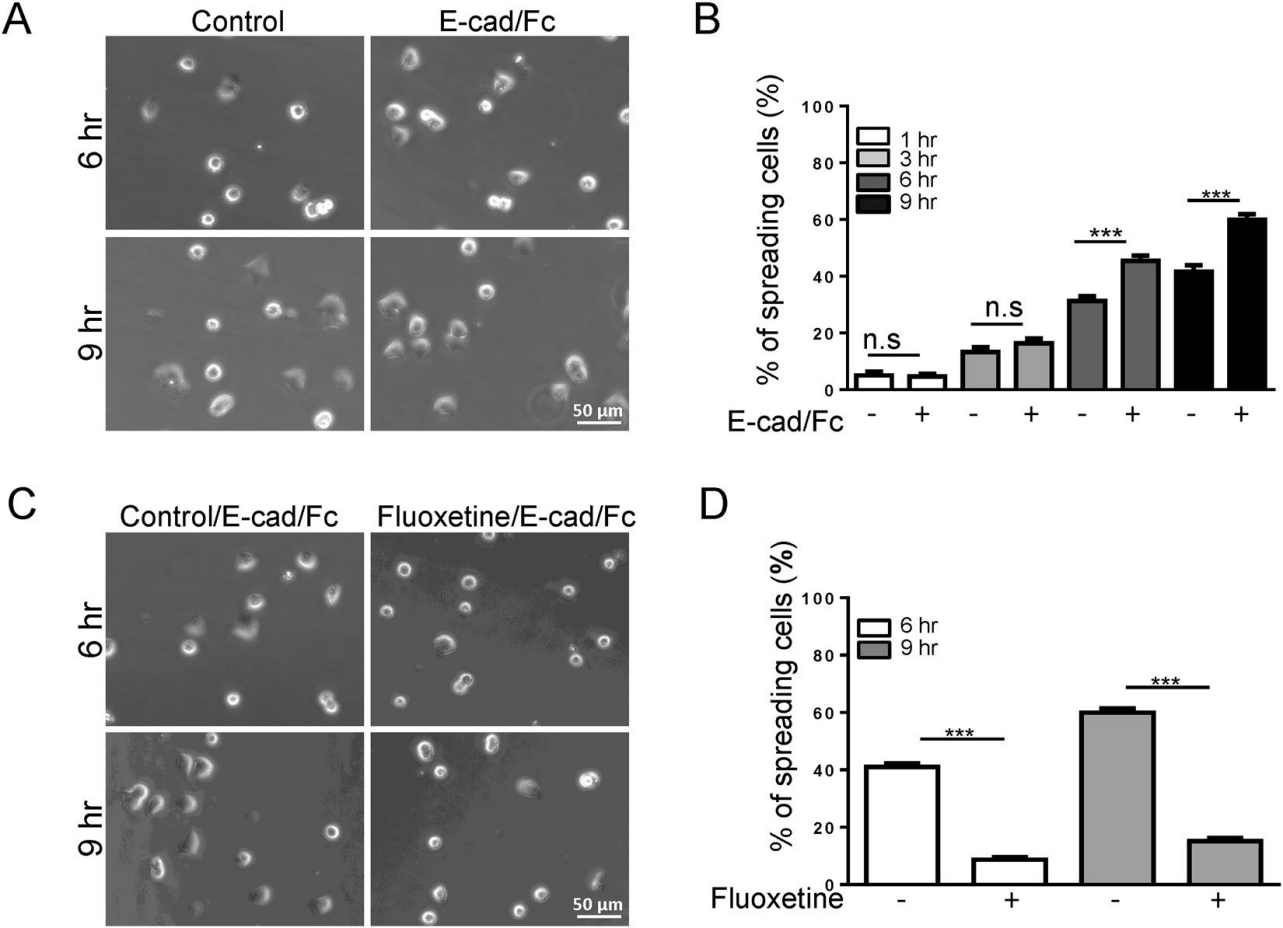
Inhibition of spreading of MIN6 cells attached to E-cad/Fc by fluoxetine. Recombinant mouse E-cad-Fc chimeric protein (5 μg/ml) was coated on glass coverslips in 4 °C for 18–24 hours, and rinsed with H_2_O before use. (**A**) MIN6 cells were seeded on control uncoated glass (control) or on glass coated with E-cad/Fc for 1, 3, 6, or 9 h. After rinsing with PBS to remove unattached cells, spreading cells were photographed by EVOS FL Auto. At least 100 cells were analyzed for each cell population. Scale bar, 50 μm (**B**) Quantitative analysis of the percentages of attached cells. Each value represents mean ± SEM from at least three independent experiments. ***p < 0.001 (**C**) MIN6 cells were seeded on glass slides coated with E-cad/Fc for 3 h or 6 h, and then treated with or without fluoxetine (30 μM) for 3 h. Cells were rinsed with PBS to remove unattached cells, and then visualized by EVOS FL Auto. At least 100 cells were analyzed for each cell population. Scale bar, 50 μm (**D**) Quantitative analysis of the percentages of spreading cells. Each value represents mean ± SEM from at least three independent experiments. ***p < 0.001.

In order to directly determine the effect of fluoxetine on E-cadherin-mediated cell adhesion, excluding the effect of other junctional proteins, recombinant E-cadherin chimeric proteins (E-cad/Fc) were employed. First, we test the effect of E-cad/Fc on β cell spreading. MIN6 cells showed a round shape when attached to the control slides; however when attached to E-cad/Fc-coated slides, they tended to flatten and to change their shape, which is characteristic of the phenomenon hereafter referred to as cell spreading (Fig. 5A). The percentage of spreading cells was significantly higher after 6 hr or 9 hr of culture on E-cad/Fc-coated slides (Fig. 5B). To further investigate the effect of fluoxetine on cell spreading, cells were seeded on E-cad/Fc-coated slides, and then incubated with or without fluoxetine. The population of round-up cells is significantly increased when MIN6 cells were treated with fluoxetine (Fig. 5C). Indeed, the quantitative data showed that fluoxetine also dramatically decreased the spreading cells (Fig. 5D). Taken together, our results showed that fluoxetine impaired E-cadherin-mediated cell adhesion, as assessed by cell morphology and spreading.

### Fluoxetine disrupts E-cadherin-mediated actin filaments

**Figure 6:**
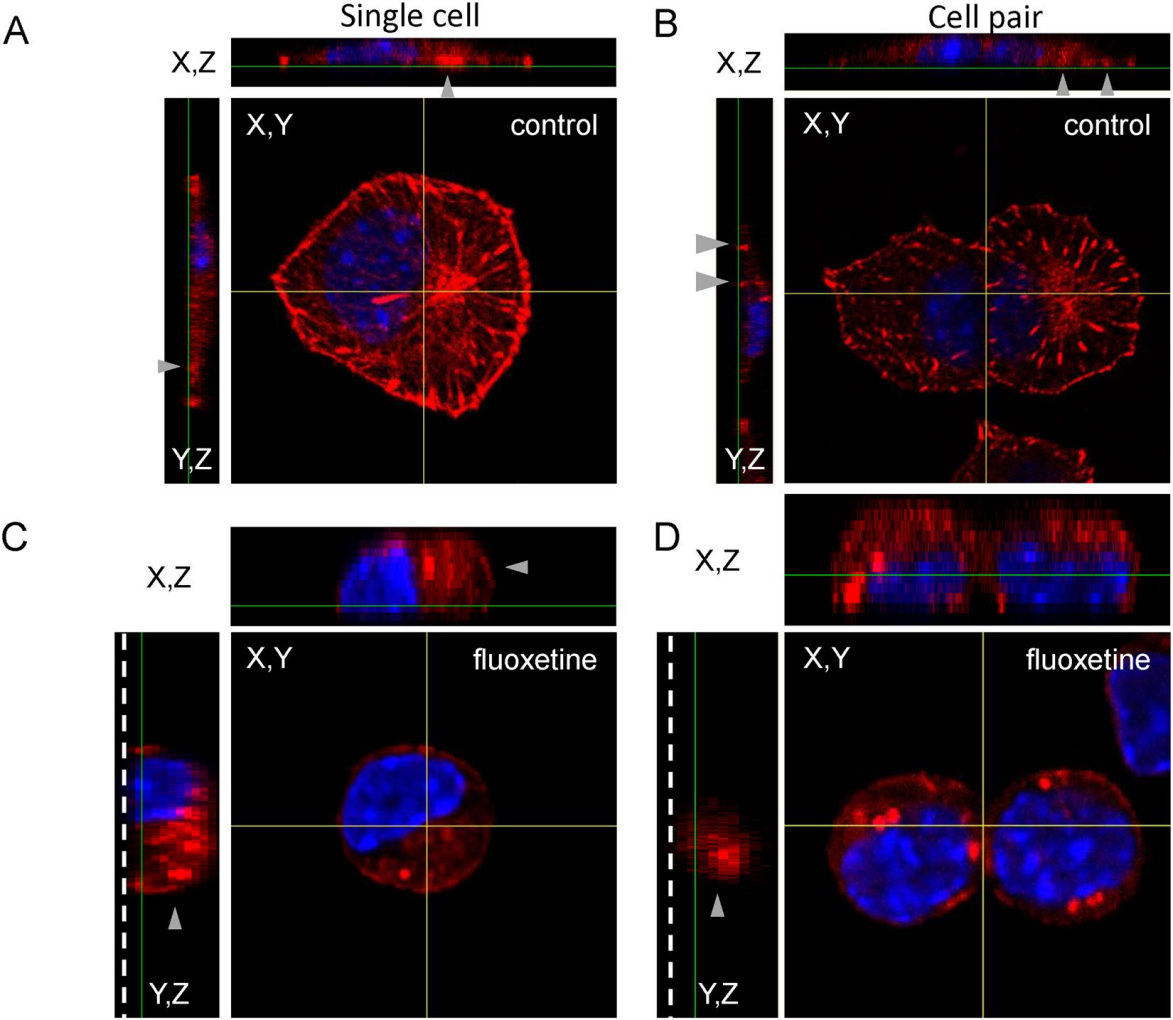
Fluoxetine alters the structure of actin filaments from MIN6 cells attached to E-cad/Fc. MIN6 cells were seeded on glass slides coated with E-cad/Fc for 6 h, and then treated with or without fluoxetine (30 μM) for 3 h. Cells were fixed and then stained with phalloidin-TRITC (red) and Hoechst 33258 (blue). In confocal image, yellow lines indicate the orientation of z-sections. The upper panel represents the x-z sections across the horizontal line. The left panel represents the y-z sections across the vertical line. The gray arrow head indicates the clustering and extension of actin filament. (**A**) Image of control single cell. (**B**) Image of control cell pair. (**C**) Image of fluoxetine-treated single cell. (**D**) Image of fluoxetine-treated cell pair.

Cortical filamentous actin (F-actin) remodeling regulates insulin granule exocytosis in β cells^42, 43^. We asked whether fluoxetine induced GSIS impairment observed in Fig. S1C, could be attributed to the impact of actin dynamics on insulin release. Staining with fluorescence-tagged phalloidin revealed that fluoxetine disrupted actin organization in cultured MIN6 cells (Fig. S3). To further confirm that cortical actin distribution was caused by disruption of E-cadherin, E-cad/Fc-coated slides and fluorescence-tagged phalloidin were employed. In control group, cortical actin was highly expressed at the cell membrane (Fig. 6A and B), but fluoxetine disrupted cortical actin structure (Fig. 6C and D).

### Fluoxetine suppresses ER calcium release and SOCE activation

**Figure 7:**
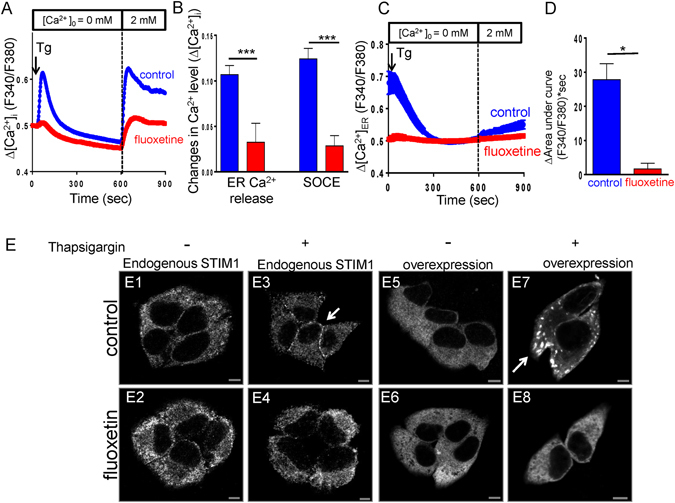
Fluoxetine inhibits the ER calcium release and SOCE activation. (**A**) Fluoxetine inhibits thapsigargin-induced SOCE activation. Mean traces of [Ca^2+^]_i_ measurement from at least 70 different cells in each experiment. The SOCE amplitude indicates the rise of [Ca^2+^]_i_ in replenishment of [Ca^2+^]_0_ from 0 to 2 mM. Arrow, adding 2 μM thapsigargin (Tg). (**B**) Quantitative analyses of Δ[Ca^2+^]_i_. Each value represents mean ± SEM of at least 70 individual cells. ***P < 0.001 (**C**) Representative measurements of ER Ca^2+^ level ([Ca^2+^]_ER_) in MIN6 cells. Mean traces of [Ca^2+^]_ER_ measurement from at least 30 different cells in each experiment. (**D**) Quantitative analyses of Δ[Ca^2+^]_ER_. Each value represents mean ± SEM of at least 60 cells. *P < 0.05 (**E**) Fluoxetine blocks STIM1 membrane trafficking. Representative confocal images of endogenous STIM1 or transiently expressed eGFP-STIM1 at control and after 5 min of exposure to thapsigargin (2 μM) in MIN6 cells treated with or without fluoxetine. Scale bar, 5 μm.

Calcium signaling plays an important role in multiple cellular functions, including insulin secretion^44^. We further studied whether fluoxetine affects calcium homeostasis in MIN6 cells, particularly focusing on ER calcium release and the SOCE activation. In fluoxetine treated cells, ER calcium release and SOCE activation were significantly suppressed, when compared with control groups (Fig. 7A and B). To determine whether the observed decrease in ER calcium release results from reduced ER calcium storage, we measured ER Ca^2+^ level by using Mag-Fura-2/AM. Our data indicated that fluoxetine significantly decreased ER calcium storage (Fig. 7C and D). STIM1 can sense the Ca^2+^ loss during ER-calcium depletion, and aggregates by translocating to plasma membrane, to finally interact and open the SOC channels leading to Ca^2+^ influx^45^. To investigate the mechanism how fluoxetine inhibits the activity of SOCE, we assessed STIM1 localization after ER Ca^2+^ depletion. Here, we showed that thapsigargin, an inhibitor of sarco/endoplasmic reticulum Ca^2+^ ATPase, induced endogenous STIM1 puncta formation with trafficking toward juxta-plasma plasma membrane (Figs 7, E3). Thapsigargin treatment also induced a similar effect on overexpressed eGFP-tagged STIM1 in control MIN6 cells (Figs 7, E7). In contrast, fluoxetine inhibited the thapsigargin-induced puncta formation and membrane trafficking of STIM1 (Figs 7, E4 and E8). These results indicated that ER calcium depletion-triggered STIM1 movement toward plasma membrane can be suppressed by fluoxetine.

### Modulating intracellular Ca^2+^ level disrupts spreading of MIN6 cells attached to E-cad/Fc

**Figure 8:**
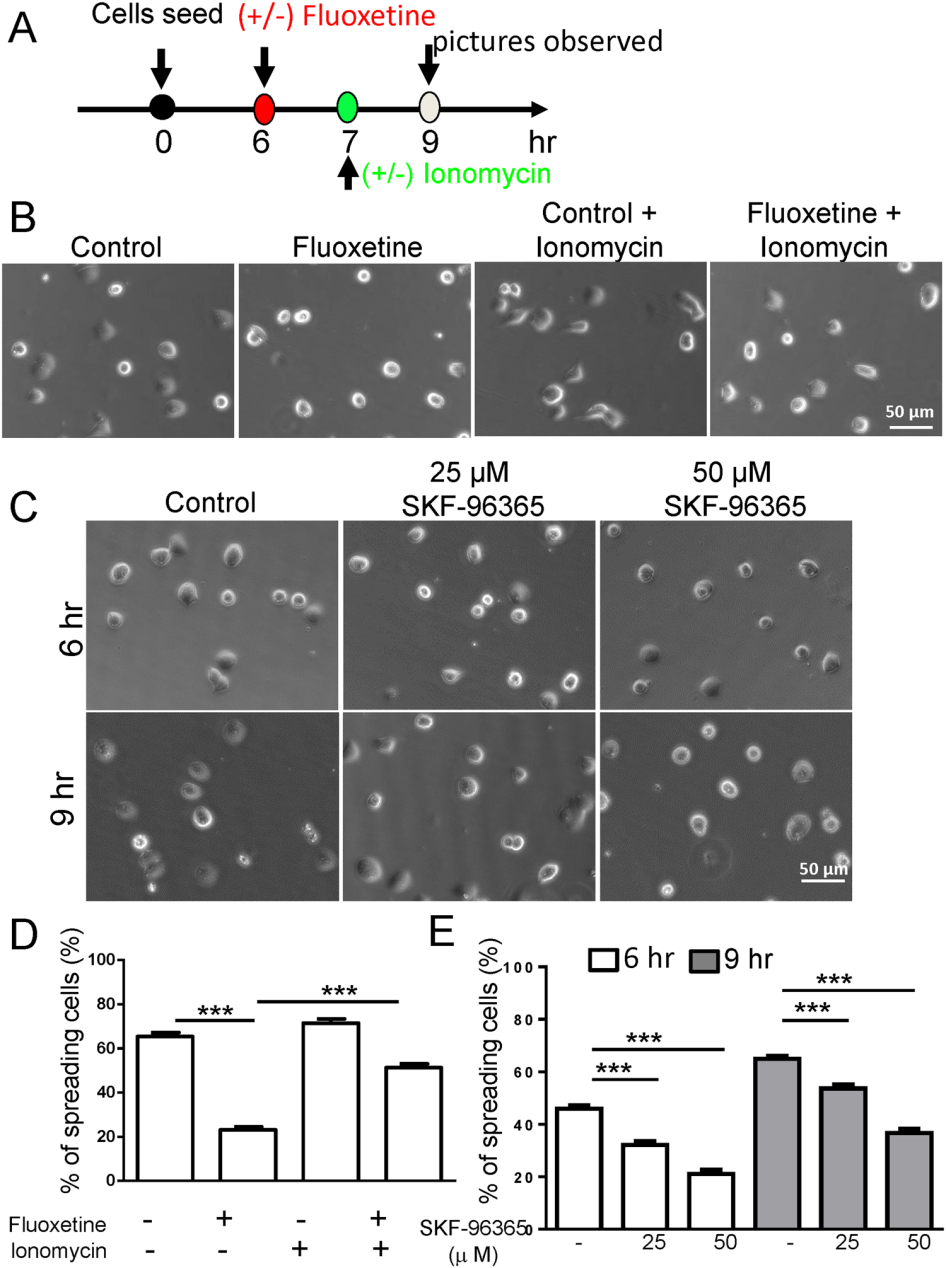
Effects of intracellular Ca^2+^ and SOCE activation on spreading of MIN6 cells attached to E-cad/Fc. (**A**) The protocol to determine whether intracellular Ca^2+^ signaling is important for cell-cell adhesion. (**B**) MIN6 cells were seeded on glass slides coated with 5 μg/ml E-cad/Fc for 6 h, and then treated with or without fluoxetine for 3 h. After one-hour incubation with fluoxetine, cells were treated with or without ionomycin (5 μM) for 2 h. Cells were rinsed with PBS to remove unattached cells, and then visualized by EVOS FL Auto. At least 100 cells were analyzed for each cell population. Scale bar, 50 μm (**C**) MIN6 cells were seeded on glass slides coated with 5 μg/ml E-cad/Fc for 4 or,7 h, and then treated with or without SKF-96365 (25 μM or 50 μM) for 2 h. Cells were rinsed with PBS to remove unattached cells, and then visualized by EVOS FL Auto. At least 100 cells were analyzed for each cell population. Scale bar, 50 μm (**D**) Quantitative analysis of the percentages of spreading cells. Each value represents mean ± SEM from at least three independent experiments. ***p < 0.001 (**E**) Quantitative analysis of the percentages of spreading cells. Each value represents mean ± SEM from at least three independent experiments. ***p < 0.001.

Because fluoxetine inhibited ER calcium release and SOCE activation, we hypothesized that intracellular Ca^2+^ might undergo certain stabilization of Ca^2+^-binding ectodomains. Here we designed a protocol to study whether intracellular Ca^2+^ is also crucial for cell-cell adhesion (Fig. 8A). Treatment with ionomycin, an ionophore used to raise the intracellular level of Ca^2+^, significantly restored the E-cad/Fc-mediated β cell spreading that were inhibited by fluoxetine (Fig. 8B and D). In addition, treatment with SKF-96365, a SOCE inhibitor, significantly decreased percentage of spreading β cells plated on the E-cad/Fc-coated slides (Fig. 8C and E). Taken together, these data suggest that fluoxetine altered intracellular Ca^2+^ level may affect E-cadherin-mediated adhesion of MIN6 cells.

## Discussion

This study highlights the impact of acute exposure to fluoxetine on inhibition of GSIS in β cells, probably via impaired E-cadherin-mediated cell adhesion and altered calcium homeostasis. This conclusion is supported by following evidence. (a) Fluoxetine disrupted cell-cell adhesion; (b) fluoxetine induced E-cadherin accumulating in Golgi apparatus; (c) fluoxetine reduced membrane-localized E-cadherin via increased endocytosis; (d) fluoxetine suppressed ER calcium release, and SOCE activation; (e) Alterations of cytosolic calcium impaired E-cadherin-mediated cell-cell adhesion.

Maintaining a tight cell-cell interaction of β cells in the islets of Langerhans is essential to form a syncytium to help the propagation and synchronization of the stimulus-secretion response from islet^46^. Indeed, studies shows that dispersed pancreatic β cells show decreased GSIS when compared to intact islets^19^. In this study, we observed definitive morphological changes in cell shape and colony phenotype upon fluoxetine treatment. Fluoxetine-treated cells lost their tightly packed arrangement and dispersed from each other with distinct intercellular gaps between adjacent cells. These changes in cell morphology may be attributed to changes in actin cytoskeleton, and E-cadherin-mediated cell-cell adhesion. In this study, we found that E-cadherin was relatively well expressed at the intercellular site of islet cells of control mice. In mice treated with fluoxetine, we observed a significant decrease in intercellular content of E-cadherin within pancreatic islets, which may be relevant to the T2DM pathogenesis. Furthermore, we also observed a significant decrease in E-cadherin in the pancreatic islets of HFD-fed mice, which is in consistent with the data of others^47^.

In this study, we defined three characteristics of cell populations from our confocal images by performing z-section from top to bottom of cells. Our results suggested that fluoxetine altered cell morphology correlated with a loss of cell-cell adhesion. The link between various cell population (combined, semi-separated, and separated) and GSIS remains to be further elucidated. Recombinant E-cadherin chimeric proteins (E-cad/Fc) were employed to directly examine the effect of fluoxetine on E-cadherin-mediated cell adhesion. Our results showed that the population of round-up cells is significantly increased when MIN6 cells were treated with fluoxetine. A previous study by Parnaud *et al*. reported that total insulin secretion was six times higher in spreading β cells compared with round β cells, suggesting that E-cadherin-mediated cell adhesion is very important for regulation of insulin secretion^18^. It has been known that insulin release from intact islets or aggregated islet cells is increased when compared with that of isolated islet cells^19^. Blockade of E-cadherin-mediated cell adhesion in pancreatic islets decreases GSIS, suggesting that loss of E-cadherin in β cells is related with impaired insulin secretion^18^. Our *in vivo* study demonstrated that pancreatic islets from mice treated with fluoxetine showed a decrease E-cadherin expression. The pancreatic islet contains different cell types (endocrine, neuronal, endothelial, mesenchymal and blood cells) that are interconnected by extracellular matrix, cell‑to‑cell adhesion molecules, cell‑to‑matrix adhesion molecules and gap junctions^48^. However, the phenomenon (various combined, semi-separated, and separated cell populations) has not been apparently observed in the pancreatic islets in this study due to the fact that many kinds of molecules and junctions are engaged in aggregated β cells and lack of technical expertise, but it is very interesting and worth of further investigation.

E-cadherin, a cell surface protein normally associated with Ca^2+^-dependent adhesive functions, is shown to mediate adhesion of β cells and to affect their insulin secretory capacity^49^. Our results revealed that fluoxetine decreased E-cadherin at the cell surface but the total amount of E-cadherin was unchanged. The cytoplasmic accumulation of E-cadherin may reflect either reduced export to the cell surface or enhanced internalization to cytoplasm. Notably, immunofluorescence staining showed that after fluoxetine treatment, cytoplasmic E-cadherin accumulated mainly in Golgi rather than ER, suggesting that E-cadherin export is retarded at Golgi. Since E-cadherin is internalized constantly through an endocytic pathway, we assume the loss of cell surface E-cadherin is also due to increased endocytosis. As expected, the confocal images revealed a significantly increased co-localization of E-cadherin with EEA1 after fluoxetine exposure. The loss of adherens junctions is one of the earliest ultrastructural changes in diabetic isletopathy^42^. Therefore, the reduction of surface E-cadherin leading to loss of tight cell-cell adhesion represented a mechanism by which fluoxetine impaired β cell function and *in vitro* GSIS.

The actin cytoskeleton, a highly dynamic structure, plays essential roles in the regulation of numerous cellular processes, such as vesicle exocytosis^50^. Cortical actin serves as a mechanical barrier, and its dissolution helps insulin granule mobilization to plasma membrane and subsequent exocytosis. In β cells, actin filaments exist as a dense web beneath the plasma membrane, and are transiently depolymerized after glucose stimulation. Interestingly, we found that most β cells were kept in round shape, and cortical actin was located on the whole cell circumference after fluoxetine treatment, which is similar to the effect of E-cadherin–neutralizing antibodies on β cell morphology^18^, suggesting fluoxetine reduces E-cadherin-mediated cell junction leading to alteration of actin filaments and cell morphology.

Fluoxetine has been shown to alter Ca^2+^ homeostasis in a variety of cell types, however, there is no study addressing this effect on pancreatic β cells. An increase in intracellular calcium ([Ca^2+^]_i_) is one of the crucial steps for insulin granule exocytosis in response to secretagogue stimulation^51^. A rise in [Ca^2+^]_i_ can also occur independently of voltage-dependent calcium channel (VDCC), by mobilization of Ca^2+^ from intracellular stores and subsequent SOCE in β cells^20^. In this study, we demonstrated for the first time that exposure to fluoxetine significantly diminished ER calcium release and inhibited SOCE activation, leading to alteration in intracellular calcium homeostasis. The ER is a key source for [Ca^2+^]_i_, particularly with regard to stimulus-secretion coupling^29^. Alterations in ER calcium handling have long been associated with islet dysfunction^28^. Here we directly measure ER calcium concentration in pancreatic β cells to clarify the role of [Ca^2+^]_i_ in the effect of fluoxetine. Our results indicated that fluoxetine altered resting [Ca^2+^]_ER_, partially, which may be correlated with ER stress, that has emerged as an important event in the pathophysiology of diabetes. Furthermore, a recent study by Isaac *et al*. has demonstrated that long-term (16 hours) treatment with 30 μM sertraline (ZOLOFT®) induces ER stress and the initiates unfolded protein response^9^. In this study, our results showed that acute fluoxetine treatment did not affect expression of 78 kDa glucose-regulated protein (GRP78), suggesting that short-term (3 h) treatment with fluoxetine did not induce an ER stress (Fig. S4). SOCE plays a potential role in Ca^2+^ entry in excitable pancreatic β cells. Recent work by Sabourin *et al*. has demonstrated that the dynamic STIM1 activation and their coupling to the plasma membrane Orai1 channels contribute to insulin secretion in rat β cells^31^. In this study, our results indicated that fluoxetine suppressed STIM1 movement toward plasma membrane, resulting in decreased SOCE activation. Another mechanism involved in the regulation of STIM1 is the phosphorylation of the protein^52^. Casas-Rua V *et al*. has described STIM1 activity is modulated by ERK1/2-dependent phosphorylation at residues Ser575, Ser608 and Ser621^53^. In this study, we found fluoxetine treatment inhibited the ERK1/2 activation and STIM1 phosphorylation at residues at Ser621 (Fig. S5). We thus propose that inhibition of ERK1/2 activation and STIM1 phosphorylation also involve in the inhibitory effect of fluoxetine on Ca^2+^ entry. Furthermore, we found that inhibition of SOCE activation is clearly associated with the reduction of β cell spreading, suggesting a functional role of SOCE in β cells. Additionally, we observed that ionomycin-induced increase of intracellular Ca^2+^ restored percentage of spreading β cells on E-cad/Fc-coated slides. Hence, calcium may be not only essential to elicit GSIS but also critical for stabilization of E-cadherin-mediated cell adhesion.

In conclusion, we demonstrated that in MIN6 cells that acute exposure to fluoxetine results in impaired GSIS, which is associated with alterations in E-cadherin and Ca^2+^ homeostasis. The present work showed the first time that the distributions of E-cadherin as well as F-actin were significantly altered in fluoxetine-treated β cells, which may be relevant to the T2DM pathogenesis in patients treated with SSRIs.

## Materials and Methods

### Cell cultures, transfection

We obtained murine insulin-secreting MIN6 cells (passages 18) from Dr. Jun-ichi Miyazaki (Osaka University Medical School) and cells were grown in Dulbecco’s Modified Eagle’s Medium (DMEM; Gibco) supplemented with 25 mM glucose, 10% heat-inactivated fetal bovine serum (FBS; Gibco), 100 U/ml penicillin, 100 μg/ml streptomycin (Gibco), 10 mM HEPEs (Gibco), 1 mM sodium pyruvate (Gibco) and 2 mM GlutaMax (Gibco). Cells were cultured at 37 °C in a humidified atmosphere containing 95% air and 5% CO_2_. Subculture and maintenance were performed as previously described^32^. Cultured cells were passaged every 3–4 days. MIN6 cells presented in this study were at passages 20–30. MIN6 cells were transiently transfected with EGFP-STIM1 by using lipofectamine 2000 (Invitrogen). At 24 h post-transfection, MIN6 cells were deprived of serum for an additional 14 h before fluoxetine incubation.

### Antibodies, chemicals, Immunoblotting

Antibodies against E-cadherin (#610182) and BiP/GRP78 (#610979) were from BD Transduction Laboratories. Antibodies against STIM1 (#5668), EEA1 (#2411), vimentin (#5541), ERK1/2 (#9102), and Phospho-ERK1/2 (#9101) were from Cell Signaling Technology. Antibodies against phosphorylation of STIM1 at Ser575, Ser608 and Ser621 were from Dr. Francisco Javier Martin-Romero, University of Extremadura, Spain. Antibodies against β-actin (clone AC-15, A5441) were from Sigma-Aldrich. Antibodies against Calnexin (GTX109669), Golgin A5 (GTX104255) were from GeneTex Inc. Antibodies against β-catenin (sc-7199), HSP90 (sc-59578) were from Santa Cruz Biotechnology, Inc. Antibodies against Calcium Pump pan PMCA ATPase (ab2825) were from abcam. Antibodies against insulin (A0564) were from Dako. SKF96365, U0126 and lonomycin were from Cayman Chemical. Fluoxetine was from Sigma-Aldrich. Mag-Fura-2/AM, Fura-2/AM and Ca^2+^-free Dulbecco’s modified Eagle’s medium (DMEM) was purchased from Invitrogen. For western blotting, cells were washed with PBS twice, and then lysed with ice-cold radioimmune precipitation assay (RIPA) buffer containing protease inhibitor cocktail (Roche Diagnostics). Protein concentrations were determined by a Bio-Rad protein assay. Equal amount of protein lysates were separated by SDS-PAGE, and then transferred to polyvinylidene fluoride (PVDF) membrane (Pall Life Science). Membranes were blocked in blocking buffer, and then incubated with primary antibodies, washed and incubated with the corresponding secondary antibodies (horseradish peroxidase-linked anti-mouse or anti-rabbit; Jackson ImmunoResearch), and visualized with western blotting luminol reagent (Santa Cruz; Millipore). Bands in the immunoblots were quantified by using ImageQuant LAS 4000 (GE Healthcare).

### Immunofluorescence, confocal microscopy, and image analyses

For immunofluorescence staining, cells were seeded on glass coverslips. After treatment, cells were fixed with 4% paraformaldehyde at 4 °C or 100% methanol at −20 °C for 5 minutes, permeabilized with 0.1% triton X-100 for 15 minutes, and blocked in 3% bovine serum albumin (Sigma-Aldrich) for 1 hour at room temperature. The cells were incubated with primary antibody in 4 °C overnight, washed, and then incubated with corresponding AlexaFluor-conjugated secondary antibodies (Invitrogen) for 1 hour at room temperature. The cells were then washed and mounted by glycerol-gelatin (Sigma-Aldrich). The images were taken by scanning confocal microscope (MPE, Olympus). The colocalization of different molecules in confocal images was pixel-by-pixel analyzed by FV-1000 software.

### Biotin surface protein isolation assay

The surface protein isolation experiments were conducted by using Pierce Cell Surface Protein Isolation Kit (Thermo Fisher Scientific). MIN6 cells grown to 60% confluence were washed twice with ice-cold PBS, and incubated with freshly prepared EZ-linked sulfo-NHS-SS-biotin in PBS at 4 °C for 30 minutes. The unreacted biotin was quenched with quenching solution. Cells were lysed and vortex for 10 seconds every 5 minutes for total 30 minutes, and then centrifuged at 12000 rpm at 4 °C for 15 minutes. Protein concentration was determined by Bio-Rad protein assay. Equal amount of protein lysates were incubated with NeutrAvidin beads at room temperature for 1 hour. The beads were extensively washed with wash buffer and PBS, and then incubated with elution buffer (50 mM DTT, 1x sample buffer) at room temperature for 1 hour. The eluent was subjected to western blotting.

### Single cell [Ca^2+^]_i_ measurement

[Ca^2+^]_i_ was measured at 37 °C with the Fura-2 fluorescence ratio method on a single-cell fluorimeter, as previously described^54^. Fura-2/acetoxymethyl ester (Fura-2/AM) were excited alternatively between 340 nm (*I*_*340*_) and 380 nm (*I*_*380*_) using the Polychrome IV monochromator (Till Photonics) and images were detected by the Olympus IX71 inverted microscope equipped with a xenon illumination system and an IMAGO CCD camera (Till Photonics). The fluorescence intensity of excitation at 510 nm was monitored to calculate [Ca^2+^]_i_ using the TILLvisION 4.0 program (Till Photonics).

### Cell adhesion assay

Recombinant mouse E-cadherin-Fc chimeric proteins containing ectodomains of E-cadherin (referred to as E-cad/Fc) were purchased from R&D Systems (Abingdon, United Kingdom). Glass coverslips were coated with or without 5 μg/ml E-cad/Fc chimeric proteins, diluted in H_2_O, and incubated at 4 °C for 18–20 hours. They were rinsed with H_2_O and air dried before use. MIN6 cells were seeded on the substrate and incubated at 37 °C for 1, 3, 6, 9 hr. At the indicated time, cells were rinsed with PBS to remove unattached cells and photographed with EVOS FL Auto (Thermo Fisher Scientific). For experiments with fluoxetine, MIN6 cells were seeded on glass slides coated with E-cad/Fc for 3 h or 6 h, and then treated with or without fluoxetine (30 μM) for 3 h. For experiments with the SOCE inhibitor, SKF-96365, MIN6 cells were seeded on glass slides coated with E-cad/Fc for 4 h or 7 h, and then treated with or without SKF-96365 (25 or 50 μM) for 2 h.

### Glucose-stimulated insulin secretion (GSIS) assay

After fluoxetine treatment, MIN6 cells were incubated for 60 min without glucose in Krebs Ringer Bicarbonate Hepes (KRBH) buffer (119 mM NaCl, 4.74 mM KCl, 2.54 mM CaCl2, 1.19 mM MgCl2, 1.19 mM KH2PO4, 25 mM NaHCO3, and 10 mM HEPES, pH 7.4, 0.1% BSA) and then induced for insulin secretion in KRBH buffer with 3.3 mM glucose (basal condition) or 16.7 mM glucose (stimulated condition) for 1 h. Secreted insulin was determined by using Mercodia ELISA kits (Uppsala, Sweden).

### Trypan blue assay

Cells were harvested at the indicated time. Cells were then washed twice with PBS, trypsinized them, centrifuged at 1200 rpm for 5 minutes, and re-suspended with 3 ml PBS. 100 μl cell suspension was mixed with 100 μl 0.4% trypan blue in isotonic solution. Unstained (viable) and stained (nonviable) cells were counted by using hemocytometer. Percentage of viable cells was calculated as follows: cell viability was the ratio of number of viable cells divided by total number of cells.

### Animals

The six-week-old C57BL/6 male mice were purchased from animal laboratory of National Cheng Kung University (NCKU) and were maintained in the pathogen-free facility of the animal Laboratory of NCKU. Experimental procedures for handling the mice were in accordance with the guidelines of the Institutional Animal Care and Use Committee (IACUC) of NCKU. All experimental protocols were approved by IACUC of NCKU, approved number 103–189. These animals were housed in a temperature- (temperature: 25 ± 2 °C; humidity: around 60–80%) and light-control environment under a 12:12 h light-dark cycle (lights on at 6:00 AM). A model of type 2 diabetes in mice was produced by high-fat diet. Eight-week-old C57BL/6 male mice were fed with an HFD containing 34.9% fat (wt/wt; 58Y1; TestDiet, Richmond, IN, USA) or a standard chow (5001; LabDiet, St. Louis, MO, USA) for up to 16 weeks. STD and HFD mice were intraperitoneally and chronically treated (4 weeks) with the SSRI fluoxetine at the active does of 20 mgkg^−1^day^−1^, as previously described^55^.

### Statistical analysis

All values were reported as mean ± SEM. Student’s paired t- test or unpaired t-test was used for statistical analyses. Differences between values were considered significant when P < 0.05.

## Electronic supplementary material

Supplementary Information
