# Supplementary material for: Selective serotonin reuptake inhibitor, fluoxetine, impairs E-cadherin-mediated cell adhesion and alters calcium homeostasis in pancreatic beta cells
Source: Sci Rep. 2017 Jun 14;7:3515. doi: 10.1038/s41598-017-03747-0 (PMC5471211; doi:10.1038/s41598-017-03747-0)
Supplement: Supplementary file 1 — Supplementary Information [file 41598_2017_3747_MOESM1_ESM.pdf]

Selective serotonin reuptake inhibitor, fluoxetine, impairs E-cadherin-mediated cell adhesion and alters calcium homeostasis in pancreatic beta cells

Huang-Yu Chang[1], Shu-Ling Chen[1], Meng-Ru Shen[1,2,3], Mei-Lang Kung[4], Lee-Ming Chuang[5,6], Yun-Wen Chen[1]

[1]Department of Pharmacology, College of Medicine, National Cheng Kung University, Tainan, Taiwan, [2]Department of Obstetrics and Gynecology, College of Medicine, National Cheng Kung University, Tainan, Taiwan, [3]Advanced Optoelectronic Technology Center, College of Engineering, National Cheng Kung University, Tainan, Taiwan, [4]Department of Chemistry, National Sun Yat-Sen University, Kaohsiung, Taiwan, [5]Department of Internal Medicine, National Taiwan University Hospital, Taipei, Taiwan,[6]Department of Medicine, National Taiwan University Medical College, Taipei, Taiwan.

Supplementary Information

Supplementary Figure S1. Fluoxetine impaired insulin secretion without affecting cell proliferation and cell viability.

Supplementary Figure S2. Fluoxetine decreases junctional E-cadherin in pancreatic islets.

Supplementary Figure S3. Fluoxetine disrupts the structure of actin filaments.

Supplementary Figure S4. Fluoxetine did not induce an ER stress.

Supplementary Figure S5. Fluoxetine inhibits ERK1/2 activation and STIM1 phosphorylation.

Supplementary Figure S6. Full length images of the cropped blots presented in main Figure 4A.

Suppl. Fig 1

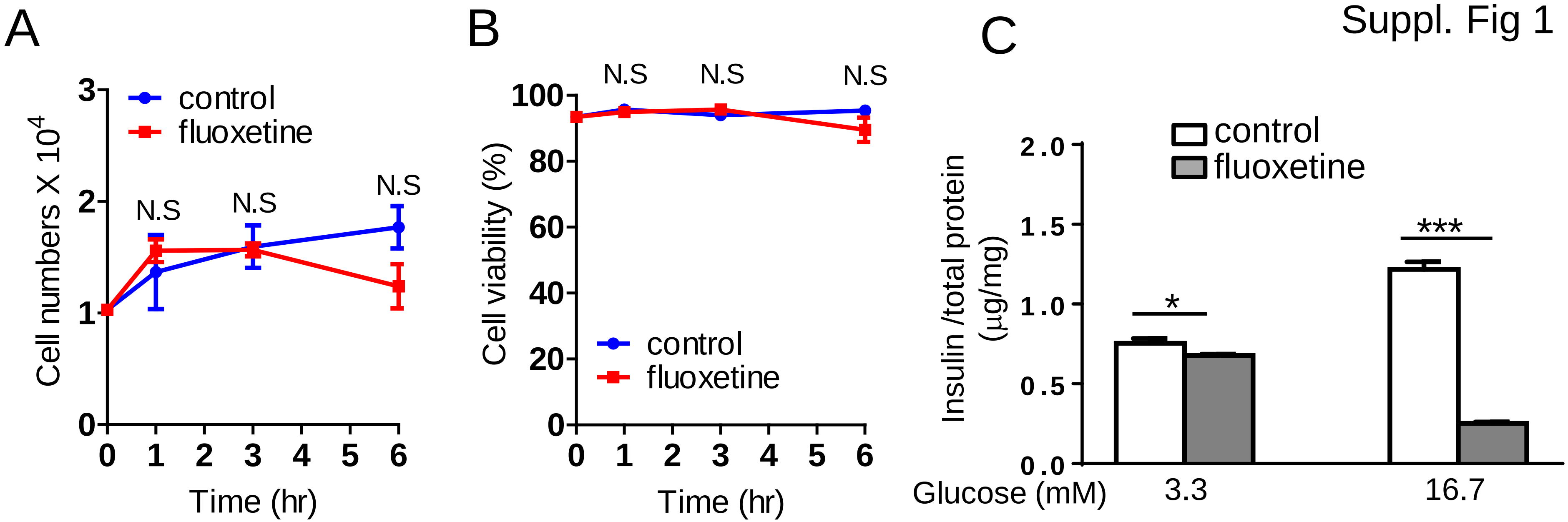

Supplementary Figure S1. Fluoxetine impaired insulin secretion without affecting cell proliferation and cell viability. Cells were seeded for 48 hours, and then were deprived of serum overnight. After indicated time of incubation with or without fluoxetine (30 μM), cells were harvested and stained with trypan blue (A) Cell proliferation of MIN6 cells treated with or without fluoxetine (30 μM). The cells were harvested at the indicated time, and living cells not stained with trypan blue were counted. Data represent the averages of three independent experiments (B) Cell viability of MIN6 cells treated with or without fluoxetine (30 μM). Cell viability was determined by trypan blue exclusion. Data was determined by trypan blue exclusion assay. Data represent the averages of three independent experiments. (C) Cells were incubated with 30 μM fluoxetine or vehicle for 3h and changed to KRBH buffer for 1 hour, cells were then stimulated with either 3.3 mM (low glucose) or 16.7 mM (high glucose) for 1 hour.The supernatant was collected and insulin content was measured by using Mercodia ELISA kits. The amounts of secreted insulin were corrected by the amounts of cell protein concentration in each well. Quantitative analysis of insulin secretion was representative data from at least three independent experiments carried out in triplicates. ***P<0.0001, Students' t-test.

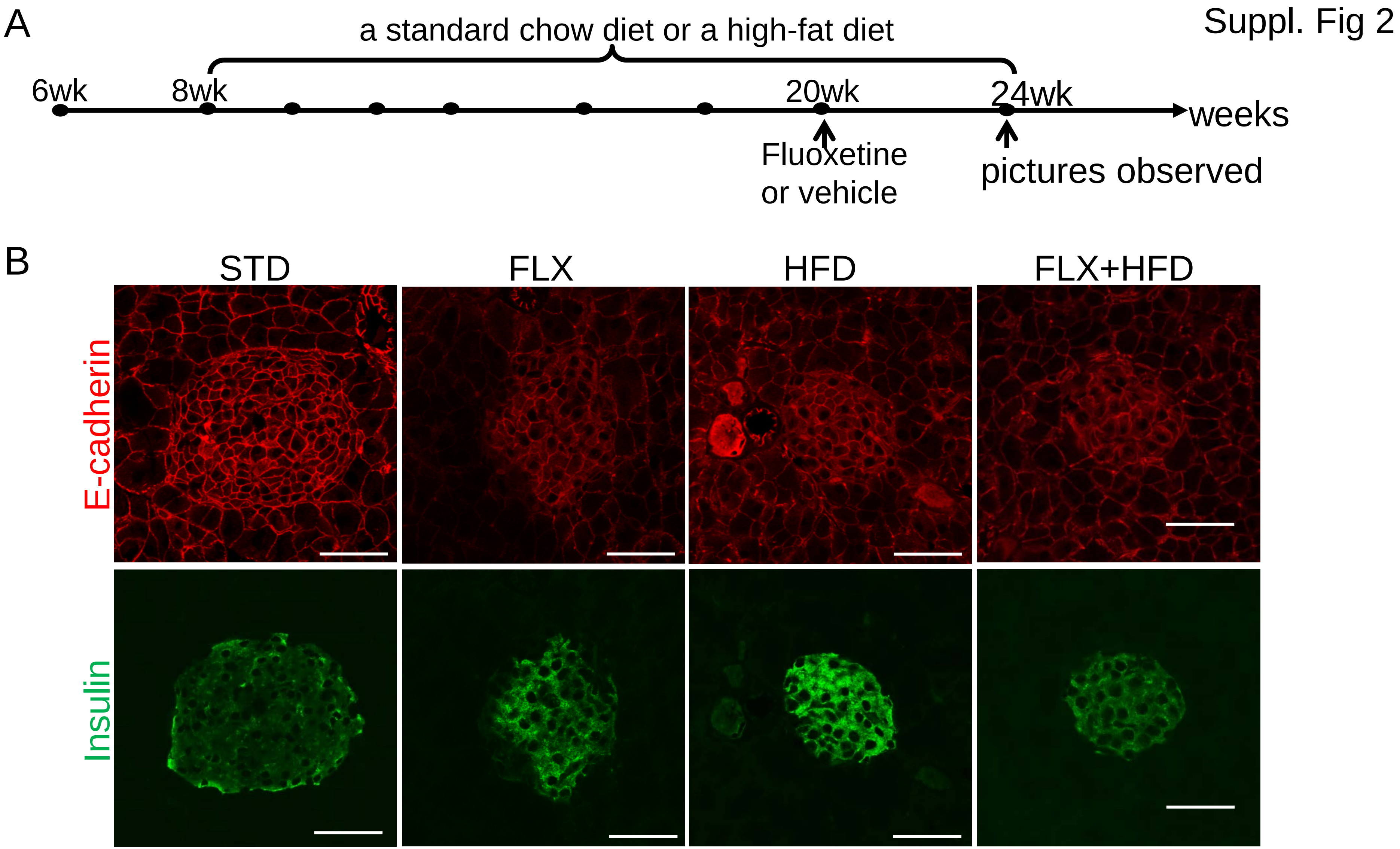

Supplementary Figure S2. Fluoxetine decreases junctional E-cadherinin pancreatic islets. (A) Experimental protocol. Mice were fed with STD or HFD for 16 weeks and received during the last 4 week a i.p. injection of vehicle (Veh) or fluoxetine (FLX) (20mg.kg^{-1}.day^{-1}) (B) The representative confocal images of E-cadherin (red) and insulin (green) in pancreatic islets of STD treated with Veh, STD treated with FLX, HFD treated with Veh, and HFD treated with FLX mice. Scale bar, 50 μm.

Suppl. Fig 3

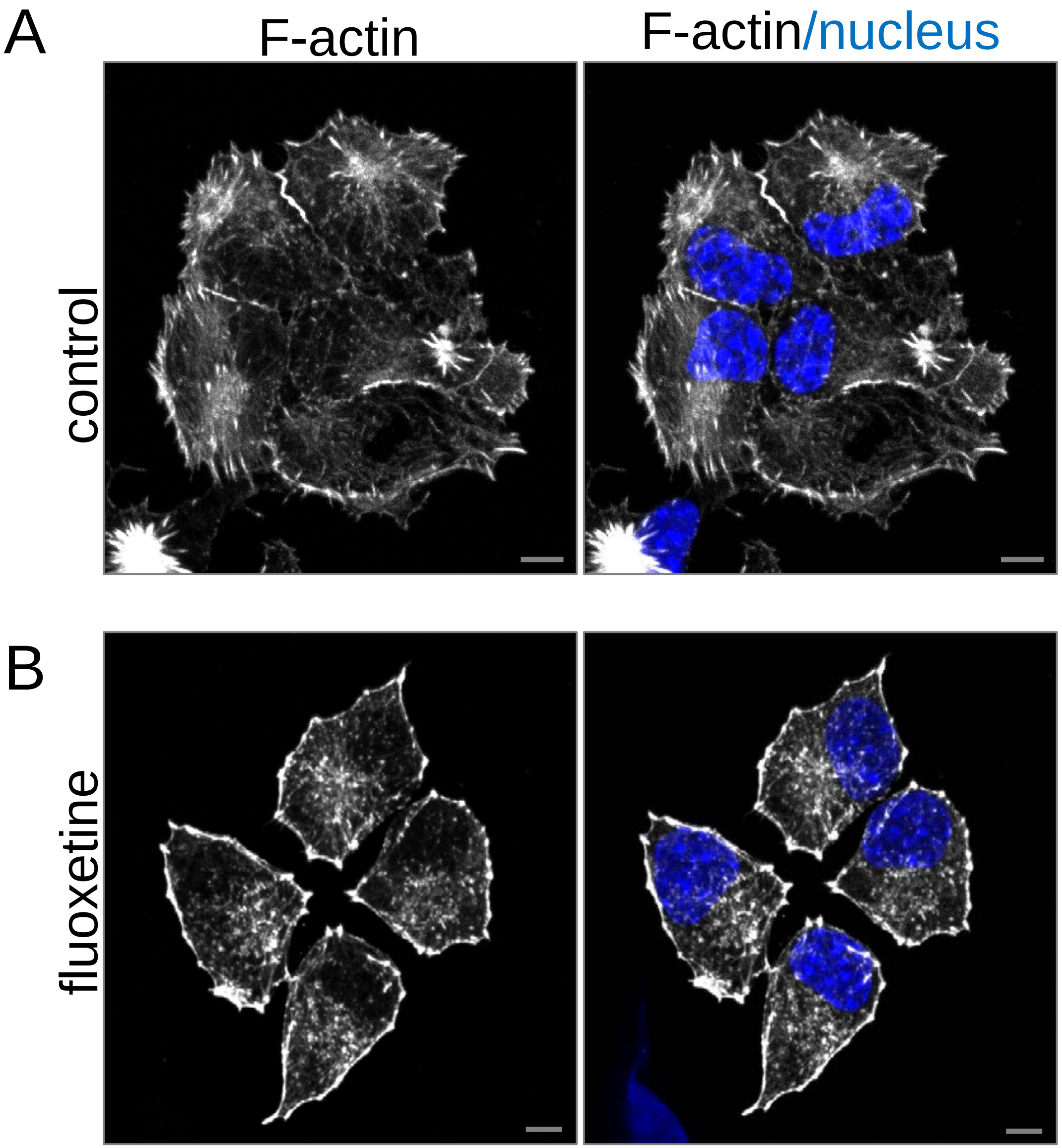

Supplementary Figure S3. Fluoxetine disrupts the structure of actin filaments. After indicated time of incubation with or without fluoxetine (30 μM), MIN6 cells were harvested and stained with phalloidin-TRITC (red) and Hoechst 33258 (blue). (A) Images of control cells. (B) Images of fluoxetine-treated cells.

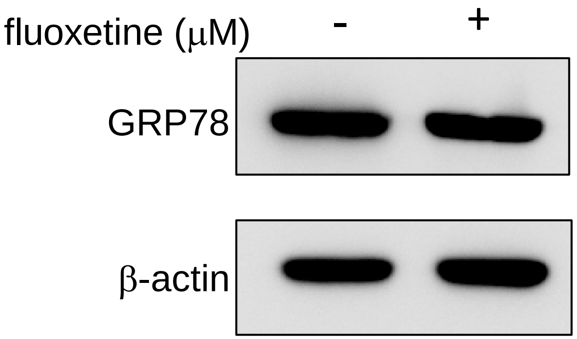

Supplementary Figure S4. Fluoxetine did not induce an ER stress. Cells were seeded for 48 hours, and then were deprived of serum overnight. After indicated time of incubation with or without fluoxetine (30 μM), cells were lysed and the extracts were electrophoresed. The GRP78 protein expression was detected by Western Blots. β-actin was used as a loading control. The representative immunoblots were from three independent experiments.

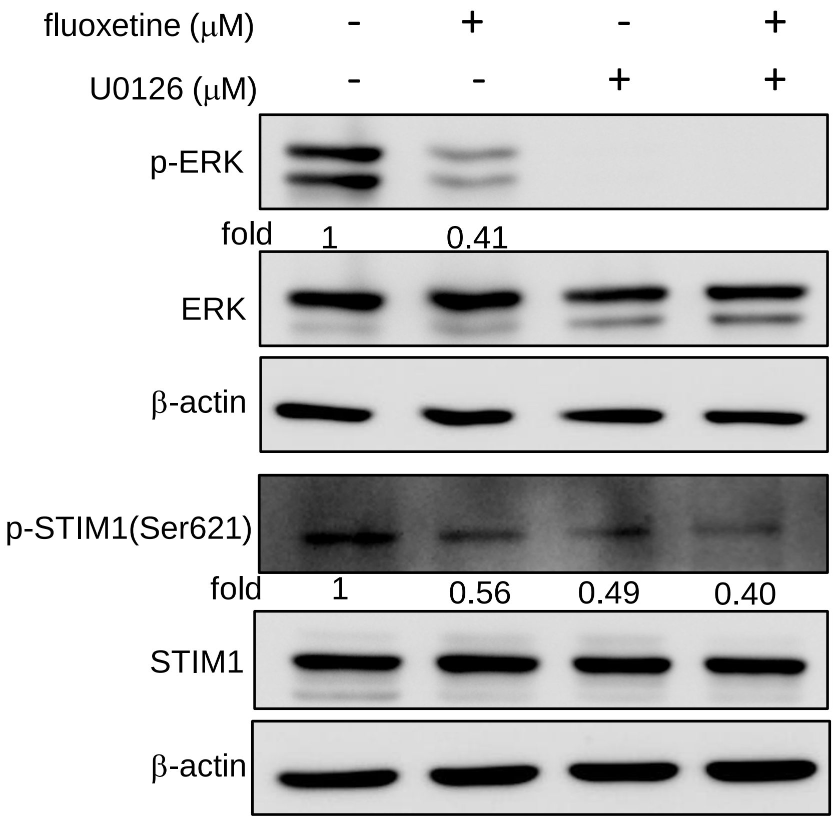

Supplementary Figure S5. Fluoxetine inhibits ERK1/2 activation and STIM1 phosphorylation. Cells were seeded for 48 hours, and then were deprived of serum overnight. After indicated time of incubation with or without fluoxetine (30 μM) and U0126 (25 μM), cells were lysed and the extracts were electrophoresed. The ERK, p-ERK, STIM1, p-STIM1 (Ser621) protein expressions were detected by Western Blots. β-actin was used as a loading control. The representative immunoblots were from three independent experiments.

Suppl. Fig 6

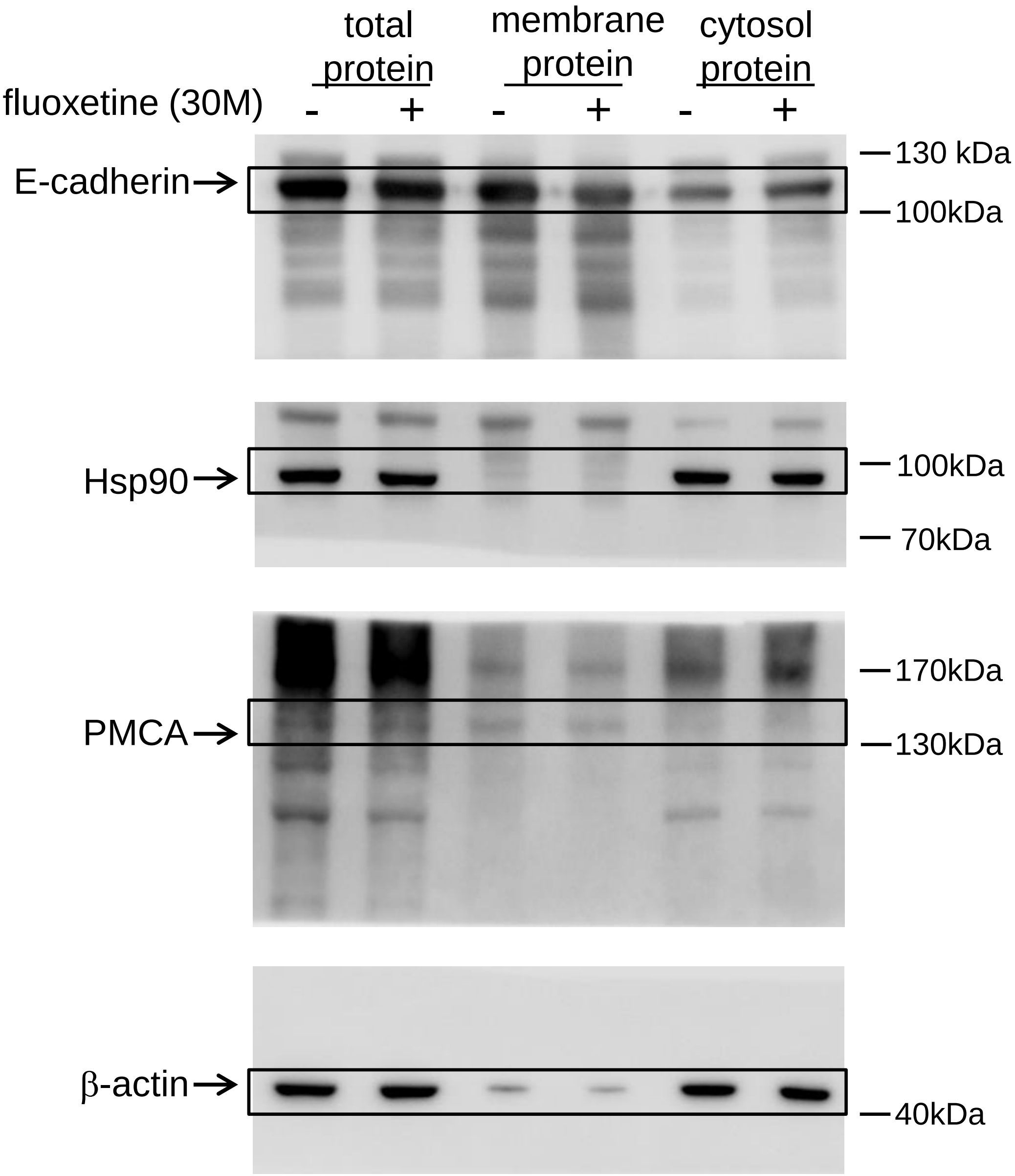

Supplementary Figure S6. Full length images of the cropped blots presented in main Figure 4A. Full length in Figure S6 showed that fluoxetine-treated cells displayed decrease E-cadherin expression at the cell surface when compared to control cells. After 3-hour incubation with or without fluoxetine (30 μM), cellular proteins were harvested by using a biotin-surface protein isolation assay, and then analyzed by western blotting. E-cadherin in total lysates, membrane fractions and cytosol fraction are shown. β-actin was used as a loading control for total protein. Hsp90 (heat shock protein 90) was used as a loading control for cytosol fraction. PMCA (calcium pump pan PMCA ATPase) was used as a loading control for membrane fraction.
